# Health economic comparison of SLIT allergen and SCIT allergoid immunotherapy in patients with seasonal grass-allergic rhinoconjunctivitis in Germany

**DOI:** 10.1186/s13601-015-0045-z

**Published:** 2015-01-21

**Authors:** Bram G Verheggen, Kirsten Y Westerhout, Carl H Schreder, Matthias Augustin

**Affiliations:** Pharmerit International, Marten Meesweg, 107 3068AV Rotterdam, The Netherlands; Stallergenes GmbH, Kamp-Lintfort, Germany; University Medical Center and Hamburg Center for Health Economics, Hamburg, Germany

## Abstract

**Background:**

Allergoids are chemically modified allergen extracts administered to reduce allergenicity and to maintain immunogenicity. Oralair® (the 5-grass tablet) is a sublingual native grass allergen tablet for pre- and co-seasonal treatment. Based on a literature review, meta-analysis, and cost-effectiveness analysis the relative effects and costs of the 5-grass tablet versus a mix of subcutaneous allergoid compounds for grass pollen allergic rhinoconjunctivitis were assessed.

**Methods:**

A Markov model with a time horizon of nine years was used to assess the costs and effects of three-year immunotherapy treatment. Relative efficacy expressed as standardized mean differences was estimated using an indirect comparison on symptom scores extracted from available clinical trials. The Rhinitis Symptom Utility Index (RSUI) was applied as a proxy to estimate utility values for symptom scores. Drug acquisition and other medical costs were derived from published sources as well as estimates for resource use, immunotherapy persistence, and occurrence of asthma. The analysis was executed from the German payer’s perspective, which includes payments of the Statutory Health Insurance (SHI) and additional payments by insurants. Comprehensive deterministic and probabilistic sensitivity analyses and different scenarios were performed to test the uncertainty concerning the incremental model outcomes.

**Results:**

The applied model predicted a cost-utility ratio of the 5-grass tablet versus a market mix of injectable allergoid products of € 12,593 per QALY in the base case analysis. Predicted incremental costs and QALYs were € 458 (95% confidence interval, CI: € 220; € 739) and 0.036 (95% CI: 0.002; 0.078), respectively. Compared to the allergoid mix the probability of the 5-grass tablet being the most cost-effective treatment option was predicted to be 76% at a willingness-to-pay threshold of € 20,000. The results were most sensitive to changes in efficacy estimates, duration of the pollen season, and immunotherapy persistence rates.

**Conclusions:**

This analysis suggests the sublingual native 5-grass tablet to be cost-effective relative to a mix of subcutaneous allergoid compounds. The robustness of these statements has been confirmed in extensive sensitivity and scenario analyses.

**Electronic supplementary material:**

The online version of this article (doi:10.1186/s13601-015-0045-z) contains supplementary material, which is available to authorized users.

## Background

According to the World Health Organization (WHO), allergic respiratory diseases have been recognized as the fourth most important chronic disease in the world and represent a major public health problem with significant quality of life (QoL) impairment [1]. With approximately one in four people presenting with clinical symptoms of allergies, the number of affected patients significantly increased in Western countries. About 90 million Europeans and 65 million Americans are affected by allergic respiratory diseases. Furthermore, 10 to 40% of patients with allergic rhinitis (AR) also have allergic asthma [2-4]. In Germany, 25% of the adult population and 21% of children suffer from AR. If untreated, AR leads to allergic asthma in 43% of the patients [5]. A lifetime prevalence of 14.8% for AR was reported [6]. Growing incidence and prevalence of allergic disorders are major reasons for the increasing need for allergen immunotherapy (AIT).

AR is an inflammation of the nasal passage that is characterized by a combination of the following symptoms: sneezing, nasal itching and/or congestion, rhinorrhoea and watery and itchy eyes [7,8]. It is caused by allergens, including proteins and glycoproteins of house dust mite fecal particles, molds, and grass or tree pollens [9].

Treatment of AR mainly consists of symptom control achieved by allergen avoidance or use of pharmacotherapy such as antihistamines. Since symptomatic medications have no long-lasting effect following discontinuation and some patients remain uncontrolled, causal treatments like AIT may be required in persistent disease and should begin as early as possible [2,10-12]. AIT interferes with basic mechanisms of allergy and alters the natural course of the disease offering long-lasting, disease-modifying and preventive effects. It is mostly used in two main types of formulations: Sublingual immunotherapy (SLIT) and subcutaneous immunotherapy (SCIT). SLIT uses an allergenic solution or tablet, applied under the tongue, which over the course of treatment reduces sensitivity to allergens. SLIT has a proven good safety profile, is convenient for patients and both adults and children can be treated at home [13]. In contrast, SCIT is administered by the doctor in form of injections.

Oralair® (the 5-grass tablet) is a SLIT tablet for treatment of seasonal, grass pollen induced AR. The active substance of the 5-grass tablet comprises freeze-dried extracts from five grasses: perennial rye (Lolium perenne), meadow (Poa pratensis), timothy (Phleum pratense), cocksfoot (Dactylis glomerata), and sweet vernal grass (Anthoxanthum odoratum) [14]. These substances correlate with the epidemiological data of patient exposure in Europe.

To provide healthcare resources within the restrictions of the healthcare system, physicians and decision-makers carefully assess the clinical benefits and economic consequences of different AIT treatments. Accordingly, a study was conducted in 2010 to assess the cost-effectiveness of the 5-grass tablet compared to Grazax® (SLIT mono-grass tablet), Alutard® (SCIT with native extracts) and symptomatic treatment for grass pollen induced AR in Germany [15]. The outcomes were based on a systematic review of the literature, a meta-analysis and the application of these clinical outcomes in a cost-effectiveness framework. Allergoids, chemically modified SCIT treatments, are a relevant competitor group in the German market. Therefore, a comparison of the 5-grass tablet versus allergoid products was performed: The meta-analysis was updated (see Additional file 1) and the existing cost-effectiveness model was extended to assess the relative effects and costs of the sublingual 5-grass tablet versus a relevant variety of subcutaneous allergoid compounds for grass pollen induced AR.

## Methods

### Model structure

**Figure 1:**
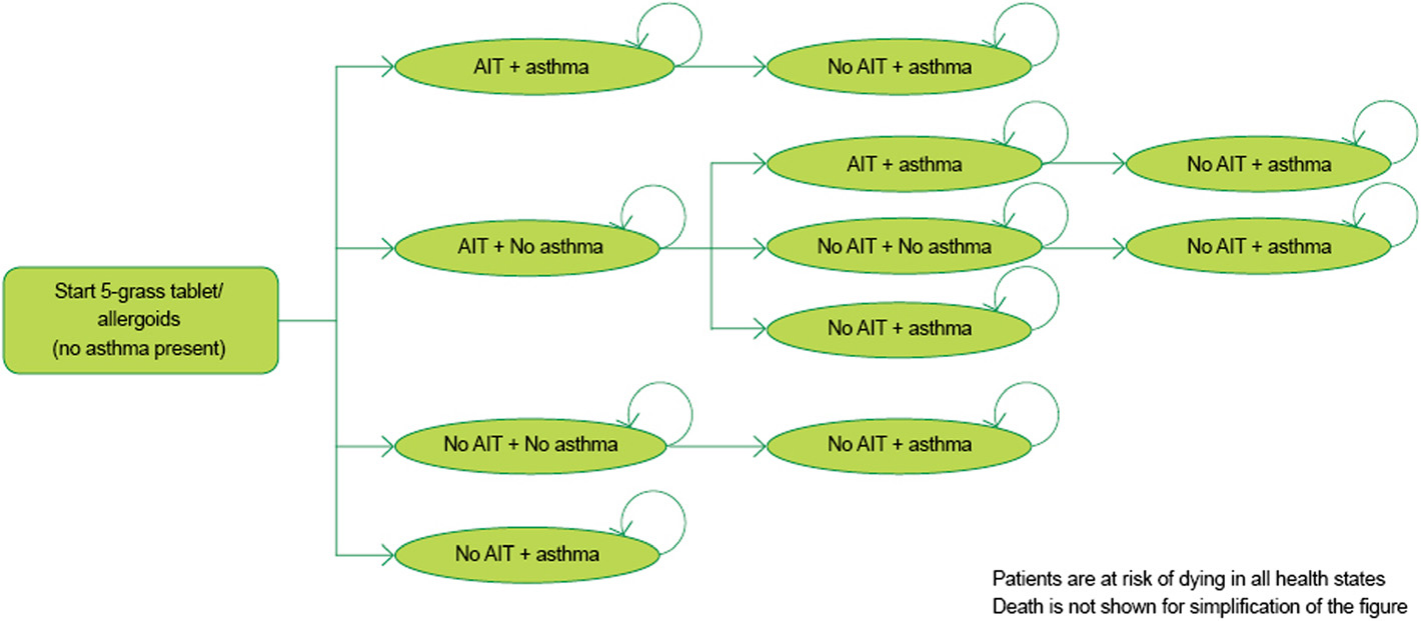
**Structure of the applied Markov model with a cycle length of one year.** Patients enter the model receiving either AIT (5-grass tablet/allergoids) or symptomatic treatment alone. AIT, allergen specific immunotherapy.

A Markov-model with a nine-year time horizon was applied to predict the distribution of patients over a number of health states over a sequence of discrete one-year time periods after receiving therapy, and subsequently to assess the associated costs and effects (Figure 1). A similar model was constructed in the cost-effectiveness analysis by Westerhout et al. [15].

Patients who entered the analysis either received AIT with the 5-grass tablet or an averaged hypothetical allergoid mix, both with optional symptomatic medication in addition, or underwent symptomatic treatment alone. The length of the pollen season was estimated at three months per year [16]. After discontinuation, solely symptomatic treatment was continued in the model. To capture both symptom control and immunologic impairment several health states were included in the model. In each state, patients could develop chronic asthma accompanied by an increased risk of dying. Every treatment arm generated corresponding symptom scores, symptom-free days, utilities and costs (see below).

### Model inputs

#### Patient population

Patients included in the model (mean age 29 years) suffered from grass pollen AR and/or conjunctivitis with a positive grass allergen-specific skin prick test and/or elevated serum grass allergen-specific IgE. Baseline characteristics of the modelled cohort reflect the average patient characteristics in the treatment arms of the included clinical trials. At entry time, none of the patients suffered from co-existing chronic asthma. Only intermittent use of ß-agonists was allowed.

#### Comparators

Within the available range of allergoid products, the most widely used products in Germany with citable references from double-blind placebo-controlled (DBPC) trials were identified. The allergoid SCITs, namely Allergovit®, Depiquick®, Pollinex® Quattro and Purethal® (all for grasses), met the search criteria. The data was then grouped and averaged as one single comparator (see also Additional file 1). As the clinical evidence for most of the individual compounds was too limited presenting only low patient numbers, grouping was indicated to obtain resilient efficacy data. Finally, symptomatic treatment (according to the trials’ standards) was included as a comparator.

#### Symptom scores and transition probabilities

In order to obtain symptom scores from all treatment arms used in the cost-effectiveness study, a literature review and network meta-analysis (type: indirect treatment comparison) were performed.

Clinical trial data on allergoid compounds published before March 2012 was retrieved from a meta-analysis performed by Di Bona et al. [17] (Table 1). In addition, the PubMed-database was searched to identify randomized control trials (RCTs) for all the included compounds published between March 2012 and May 2013 (Table 1). The literature review and indirect comparison is detailed in the Additional file 1. AIT is typically administered during three consecutive seasons, and it was assumed that drug effects during these three seasons could be viewed as independent. Therefore, the symptom scores reported in clinical trials during the on-treatment years were pooled in the indirect comparison, resulting in one single value per treatment. Rhinoconjunctivits Total Symptom Scores (RTSS) values were: 5-grass tablet 3.26, allergoid mix 3.64, and symptomatic treatment 4.47. Rates for discontinuation, developing asthma and mortality as implemented in the model are shown in Table 2. It was further assumed that these symptom score values remain constant during the post-treatment period in the model.

**Table 1:** **Randomized controlled trials (RCTs) for the 5-grass tablet and the allergoid mix**

	**RCT**
**5-grass tablet**	Didier 2007 [18]
	Wahn 2009 [19]
	Didier 2011 [20]
	Cox 2012 [21]
**Allergoid mix**	Pfaar 2012 (Depiquick) [22]
	Corrigan 2009 (Allergovit) [23]
	Drachenberg 2001 (Pollinex) [24]
	Du Buske 2011 (Pollinex) [25]
	Brewczynski 1999 (Purethal) [26]

**Table 2:** **Transition probabilities applied in the model**

**Parameter**		**Probability**	**Reference**
Discontinuation SLIT (5-grass tablet)	Year 1	0.29	[27]
	Year 2	0.28	[27]
Discontinuation SCIT (allergoid mix)	Year 1	0.41	[27]
	Year 2	0.34	[27]
Developing asthma with symptomatic treatment		0.0046	[28]
Relative risk developing asthma (AIT vs. symptomatic treatment)		0.505	[29,30]
All population mortality*		0.00046	[31]
Asthma mortality*		0.00069	[32]

*Probabilities represent mortality at start of the analysis (29 years).

#### Utility values

For clinical trials and cost-effectiveness studies, the Rhinitis Symptom Utility Index (RSUI) has been developed. RSUI represents a preference-based utility index for rhinitis symptoms using standard gamble and visual analogue scale (VAS) [33]. Using this scoring index, symptom severity can be converted to utility. Patients’ QoL values were further determined by patient’s age and co-existing asthma during the pollen season [34,35].

#### Costs

The analysis was conducted from the German payer perspective, including payments of the SHI and additional payments by insurants. All AIT agents were used for a maximum time period of three years. Costs for the 5-grass tablet administered once daily were based on usage of seven months per year (pre-seasonal and co-seasonal) in the base case analysis [14]. For the allergoid mix (see above), a weighted average cost was calculated based on German market shares (December 2011). For all allergoid compounds, one package was sufficient to treat a patient before and/or during the season [36-38].

All pharmaceutical costs reflect SHI and consumer (co-)payments. Additionally, costs for symptomatic medication were calculated by multiplying the costs of loratadine and budesonide by the number of actuations. The number of tablets and puffs used during the season for the AIT and symptomatic treatment arm presented in Bachert et al. [39] were adjusted for an average pollen season’s length of three months (Table 3).

**Table 3:** **Treatment costs**

**Model parameter**		**Value**
***Costs of immunotherapy treatment over a 3-year treatment period***		
5-grass tablet		€ 2,100.10
Allergoid mix		€ 1,449.60
***Seasonal costs and symptomatic medication per season***		
Loratadine	AIT	€ 5.14
	Symptomatic treatment	€ 7.54
Budesonide	AIT	€ 2.19
	Symptomatic treatment	€ 3.83
***Weighted average costs of resource use***		
Specialist visit 10 minutes		€ 13.29
Additional costs visits > 10 min		€ 3.69
AIT injection		€ 5.11
Diagnostic tests		€ 20.61
***Costs of asthma indexed for 2013***		
2010		€ 175.00
2013		€ 186.30

Apart from pharmaceutical costs, the model included costs for specialist visits, treatment administration, diagnostics and asthma. In Germany, costs for health care are paid in four different settings: Public sector (90%), which is separated in lump sums (80%) [40], ambulatory hospital setting (15%) [41], and visits to a doctor who is remunerated by the schedule of port fees and charges for doctors (Gebührenordnung für Ärzte, GOÄ) instead of the general assessment standard (Einheitlicher Bewertungsmaßstab, EBM) (5%) [39]. The last setting is private health care insurance (10%) [42]. Table 3 presents the average resource cost of all four remuneration options.

The number of specialist visits associated with the use of the allergoid mix was estimated based on a weighted average of the number of injections each year taken from the Summary of Product Characteristics (SmPC). 5-grass tablet patients were assumed to see their doctor every three months during treatment for optimal adherence [43]. All patients on immunotherapy were estimated to have one visit for a diagnostic test in the first year. After three years of AIT treatment all patients were expected to have 1.9 visits per year [44]. Finally, three references were used to derive the cost for asthma [45-47] and are presented in Table 3.

#### Model outcomes

Model outcomes were calculated and presented as total and incremental (un)discounted QALYs and costs. Then, a cost per Quality Adjusted Life year (QALY) was derived for the 5-grasss tablet and the allergoid mix. In accordance with the guideline for pharmacoeconomic research in Germany, discounting was applied at 3% per year for both costs and effects [48].

#### Sensitivity analyses

Deterministic and probabilistic sensitivity analyses (PSA) were performed as well as a number of scenarios to assess the influence of uncertainty of input parameters. To identify the main drivers of the model outcomes, all uncertain parameters were placed separately at their outer limits of their 95% confidence intervals within the deterministic univariate sensitivity analyses.

To constitute the uncertainty around the predicted incremental costs and effects, 1.000 simulations were performed in the PSA by simultaneously changing the parameters by random draws from their estimated distributions. For transition probabilities and utility values, beta distribution was applied. Normal distributions were used in case of risk estimates, and treatment efficacy of the 5-grass tablet, and allergoid mix (standardized mean difference values, SMDs). Gamma distributions were applied for healthcare costs and the duration of the pollen season. If the publication did not provide information on variance, the standard error was supposed to vary 20% around the mean value.

A scatterplot and an acceptability curve were designed estimating the 95% confidence intervals around incremental model outcomes and the probability of the 5-grass tablet being cost-effective versus its comparators at a given willingness-to-pay (WTP) threshold per obtained QALY. Since the effects of assumptions and choices may not be completely captured by the sensitivity analyses, a number of scenario analyses were carried out.

For the base case analysis, an assumption was made on the distribution of patients on the various remuneration pathways. Due to variations in this respect, costs for specialist visits, treatment administration, and diagnostics were calculated based on lump sum payments (scenario 1) and the ambulatory setting (scenario 2).

In another scenario, costs were obtained from the societal perspective, incorporating indirect costs. Labour hours missed [39] over one season due to specialist visits e.g. were multiplied by the cost of one labour hour (€ 30.70) [42,49]. In a fourth scenario, the utility data were based on a different literature source. Utility values measured with the EQ-5D associated with another mono-grass tablet (Grazax®) (0.976) and symptomatic treatment (0.947) were also available from a cost-effectiveness study [39]. As only data for the mono-grass tablet existed, AIT agents considered in the current analysis (5-grass tablet/allergoid mix) were assumed to all be associated with the same utility value of 0.947.

Since the length of the pollen season varies in different geographical areas, in scenario 5 a shorter duration of two months was evaluated.

## Results

### Base case analysis

#### Effects

The results after nine years indicate higher total and incremental effects (QALYs) for the 5-grass tablet, for both discounted and undiscounted values (Table 4). This is based on the better efficacy of the 5-grass tablet in terms of RTSS compared with the allergoid mix. Additionally, AIT with the 5-grass tablet results in a lower total number of incidental asthma patients compared to symptomatic treatment.

**Table 4:** **Base case results**

**Overview of base case results comparing SLIT allergen and SCIT allergoid immunotherapy**						
**Discounted and undiscounted total QALYs per treatment after a time horizon of 9 years**						
	**Discounted**	**Undiscounted**				
5-grass tablet	7.316	8.207				
Allergoid mix	7.280	8.166				
Symptomatic tx	7.235	8.116				
**Absolute and relative distribution of total undiscounted costs over individual cost components after 9 years**						
	**5-grass tablet**		**Allergoid mix**		**Symptomatic**	
	Cost	Perc.	Cost	Perc.	Cost	Perc.
Visits (e.g. Dermatologist)	€ 267	15.0%	€ 299	22.9%	€ 227	62.4%
Injection/control	€ 4	0.2%	€ 69	5.2%	€ 0	0.0%
Diagnostics	€ 21	1.2%	€ 21	1.6%	€ 0	0.0%
Treatment costs	€ 1,381	77.7%	€ 809	61.8%	€ 0	0.0%
Other drugs costs	€ 82	4.6%	€ 86	6.6%	€ 102	28.1%
Asthma	€ 23	1.3%	€ 25	1.9%	€ 34	9.5%
Total	€ 1,778	100%	€ 1,308	100%	€ 363	100.0%
**Discounted and undiscounted total costs per treatment and incremental costs after a time horizon of 9 years**						
	**Discounted**		**Undiscounted**			
	Total	Inc. vs. 5-grass tablet	Total	Inc. vs. 5-grass tablet		
5-grass tablet	€ 1,707		€ 1,778			
Allergoid mix	€ 1,249	€ 458	€ 1,308	€ 470		
Symptomatic tx	€ 322	€ 1,385	€ 363	€ 1,415		
**Discounted and undiscounted incremental costs and ICERs after a time horizon of 9 years**						
	**Inc. Costs**	**Inc. QALYs**	**ICER**			
Discounted						
5-grass tablet vs. Allergoid mix	€ 458	0.036	€ 12,593			
5-grass tablet vs. Symptomatic tx	€ 1,385	0.081	€ 17,007			
Undiscounted						
5-grass tablet vs. Allergoid mix	€ 470	0.041	€ 11,576			
5-grass tablet vs. Symptomatic tx	€ 1,415	0.090	€ 15,635			

#### Costs

AIT treatment with the allergoid mix show a substantial use of health care services, as injections need to be administered by the specialist. Analyzing the absolute and relative distribution of the total costs over separate cost components, AIT treatment costs were found to be the main cost drivers (61–78% of the total amount), followed by cost for dermatologist visits (Table 4). Discounted and undiscounted values of total and incremental costs are displayed in Table 4.

#### Incremental cost effectiveness ratio (ICER)

Table 4 presents estimates of incremental costs and effects after nine years of treatment and follow-up, both discounted and undiscounted. These incremental values result in a cost-effectiveness ratio of the 5-grass tablet relative to symptomatic treatment of € 17,007 per QALY. For the 5-grass tablet vs. allergoid mix the ICER is € 12,593 per QALY.

### Sensitivity analyses

For the comparisons of the 5-grass tablet versus allergoid mix, extensive deterministic univariate sensitivity analyses and one probabilistic multivariate sensitivity analysis have been conducted, as well as a number of scenarios.

#### Deterministic univariate sensitivity analyses

**Figure 2:**
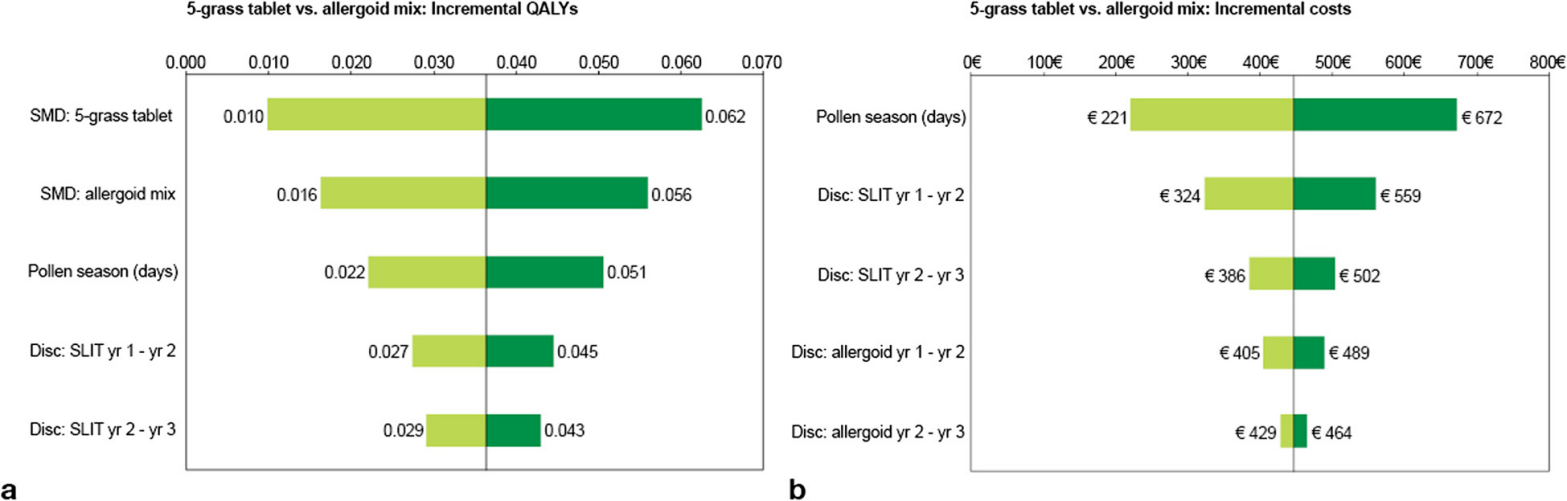
**Results of the deterministic univariate sensitivity analyses. a)** Tornado graph representing outer limits of incremental QALYs for the 5-grass tablet vs. allergoid mix and **b)** Tornado graph representing outer limits of incremental costs for the 5-grass tablet vs. allergoid mix.

Incremental QALYs resulting from the calculations are mainly sensitive to changes in efficacy estimates, because these are directly linked to utilities from the RSUI (Figure 2a). The length of the pollen season shows an influence on the incremental QALYs. The longer the season, the more QALYs gained for the 5-grass tablet versus its comparators due to the higher RSUI for the 5-grass tablet. Furthermore, changes in discontinuation rates have a slight effect on the results. When comparing the 5-grass tablet vs. the allergoid mix, the incremental costs are most sensitive to the length of the pollen season, as the cost for 5-grass tablet depends on a season’s duration (Figure 2b). Furthermore, incremental costs are most sensitive to probabilities of immunotherapy discontinuation. In general cost outcomes are only mildly influenced by parameter uncertainty, as immunotherapy treatment is the main cost driver behind the results on incremental costs.

#### Probabilistic multivariate sensitivity analysis

**Figure 3:**
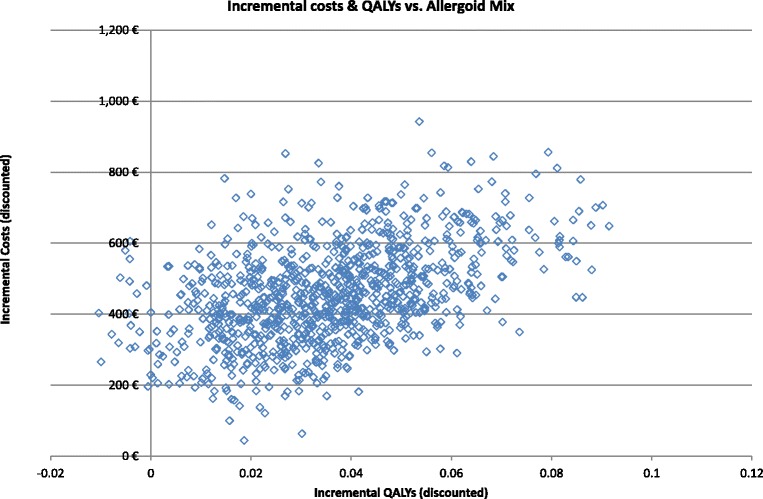
**Scatter plot presenting the incremental costs and incremental QALYs generated in the conducted multivariate sensitivity analyses for the 5-grass tablet vs. allergoid mix.**

**Figure 4:**
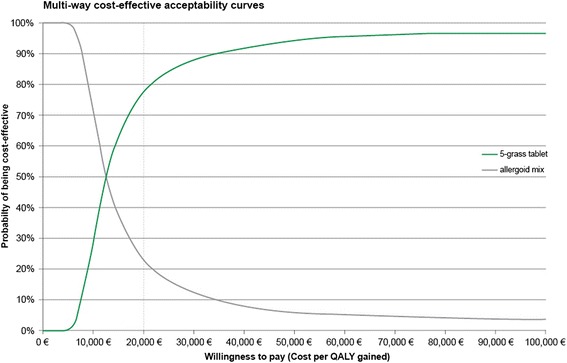
**Cost effectiveness acceptability curves, showing the probability of the 5-grass tablet and allergoid mix being most cost-effective at a range of willingness to pay thresholds.**

The joint uncertainty around the incremental costs and QALYs are displayed in Figure 3. The percentage of simulations which predicts positive effects and higher costs is estimated at 98% when the 5-grass tablet is compared to the allergoid mix. The 95% confidence intervals for the incremental QALYs are 0.002 to 0.079 for 5-grass tablet vs. allergoid mix. The 95% confidence intervals for costs are € 192 to € 718. Figure 4 present the acceptability curves of the 5-grass tablet and the allergoid mix. At a willingness-to-pay threshold of € 20,000, the probability of the 5-grass tablet being the most cost-effective treatment is predicted at 76%.

#### Scenario analyses

In the first two scenario analyses, healthcare costs are calculated by lump sums or 1 fold GOÄ (Gebührenordnung für Ärzte, remuneration catalogue for physicians) prices. In contrast to the first scenario, the second one did remunerate for extra services performed by the physician. As a result, incremental costs of the 5-grass tablet increased (lump sums) and decreased (1-fold GOÄ) versus SCIT allergoids, since injections correspond to extra visits (Table 5).

**Table 5:** **Results of the scenario analyses**

**Scenario**	**ΔQALY**	**ΔCosts**	**ICER**
***Base case values***			
- 5-grass tablet vs. allergoid mix	0.036	€ 458	€ 12,593
- 5-grass tablet vs. symptomatic tx	0.081	€ 1,385	€ 17,007
***Scenario analysis 1: 100% Lump sums***			
- 5-grass tablet vs. allergoid mix	0.036	€ 546	€ 15,025
- 5-grass tablet vs. symptomatic tx	0.081	€ 1,362	€ 16,723
***Scenario analysis 2: 100% Public to private (ambulatory)***			
- 5-grass tablet vs. allergoid mix	0.036	€ 305	€ 8,400
- 5-grass tablet vs. symptomatic tx	0.081	€ 1,414	€ 17,367
***Scenario analysis 3: Societal perspective***			
- 5-grass tablet vs. allergoid mix	0.036	€ 339	€ 9,327
- 5-grass tablet vs. symptomatic tx	0.081	€ 784	€ 9,634
***Scenario analysis 4: Changing utilities***			
- 5-grass tablet vs. allergoid mix	0.026	€ 458	€ 17,531
- 5-grass tablet vs. symptomatic tx	0.131	€ 1,385	€ 10,557
***Scenario analysis 5: Shorter pollen season***			
- 5-grass tablet vs. allergoid mix	0.024	€ 266	€ 10,966
- 5-grass tablet vs. symptomatic tx	0.055	€ 1,198	€ 21,918

By including productivity losses as performed in scenario 3, incremental costs of the 5-grass tablet versus the allergoid mix and symptomatic treatment decreased, because treatment with AIT is associated with fewer hours lost from work [50]. The study of Peterson et al. revealed that sick days were reduced from 3.7 to 1.2 days by the treatment with AIT [50]. Utility data from Bachert et al. [39] were incorporated into the economic model as input parameters for QoL (scenario 4). Differences in QALY gain between the 5-grass tablet and the allergoid mix are due to differences in discontinuation rates. Decreasing the length of the pollen season in scenario 5 affected both incremental costs and effects. Incremental costs decreased as the 5-grass tablet was administered for a shorter duration. Incremental QALYs decreased as the benefits of the higher RSUI for the 5-grass tablet was applied for a shorter duration.

## Discussion

The primary objective of the present analysis was to estimate the costs and effects of the 5-grass tablet in comparison to an average mix of allergoid compounds in patients with seasonal grass pollen-induced AR. The importance for a cost-effective treatment of AR results from the increasing prevalence of AR in conjunction with a lower QoL and reduced absenteeism and/or presenteeism at work. In that respect, the base case model outcomes can be considered conservative as productivity losses were not included. Time away from work due to AR was measured in a clinical trial [39] and used for productivity losses in a scenario analysis. Including these time losses result in higher productivity costs for SCIT allergoid comparators.

The base case analyses revealed that point estimates for cost-effectiveness are considered to be commonly acceptable in Western countries. However, uncertainty surrounding the model predictions exists. Results were especially sensitive to changes in discontinuation rates and efficacy estimates over the season, which was shown by univariate sensitivity analyses. This is due to the fact that AIT is the main cost component and symptom severity is directly linked to QoL. Furthermore, since the cost of the 5-grass tablet is dependent on a season’s duration, incremental outcomes were sensitive to the length of the pollen season as well.

Optimally, a direct evaluation of several treatment options in a clinical trial is used to examine relative efficacy data. Due to the lack of direct comparative data analyzing AIT, an indirect comparison was used to compare the efficacy of relevant treatment options in one pharmacoeconomic evaluation. In the indirect comparison symptomatic treatment was used as the common comparator. A fixed effects model was used applying inverse variance weighting to correct for study sample size. Studies with a small patient population and high uncertainty around the means attained a lower weight and therefore had less impact on the pooled value. In addition, inclusion criteria for the studies were very similar. Although the chosen methodology has limitations, the determination of comparative efficacy is more accurate by combining published evidence of multiple trials in one meta-analytical framework than using data from individual trials alone.

One of the most important model assumptions relates to the extrapolation of the treatment effect. Drug treatment was administered for three years and the effect observed during treatment was extrapolated until the end of the ninth year. Observations from a number of studies serve as the basis for this assumption, showing sustained efficacy after three years of treatment [51]. In addition, the long-term effects of the 5-grass tablet are currently investigated in a five-year clinical trial. Sustained efficacy of the pre- and co-seasonal 5-grass tablet therapy have lately been demonstrated in the post-treatment year (fourth year) [52], while this evidence has not been demonstrated for allergoids. Nevertheless, a conservative approach was taken in that the on-treatment symptom score for allergoids was also assumed to remain constant during the post-treatment phase.

Published cost-effective analyses on grass allergens usually project the results of the comparisons over a similar time horizon of nine years [34,39,53].

Prevention of asthma and new sensitizations in patients with AR are further crucial objectives of AIT. Selected allergen extracts have shown a persistent long-term effect on clinical symptoms after termination of treatment and long-term, preventive effect on later development of asthma in children with seasonal rhinoconjunctivitis [54]. The risk ratio of developing asthma with AIT treatment partially grounds on a study with SCIT and another SLIT compound [29,30]. The latter has not shown extensive evidence of testing dosing schemes to achieve optimal effects as the investigated 5-grass tablet may be underdosed and might not show optimal results.

This analysis excludes the rare occurrence of hospitalizations and the examination of consequences of potential side effects such as itching or swelling. Side effects observed with SLIT tablets (i.e. 5-grass tablet) are, in most cases, mild and reversible, appearing mostly only during the early phase of treatment [14]. Although SCIT is associated with such mild adverse events as well, severe systemic reactions, potentially resulting in an anaphylactic shock have been observed [55]. However, including these rare systemic adverse events would not influence QoL or healthcare costs in a significant manner on a population level.

Comparable health economic comparisons are rare in the literature. An economic evaluation assessed the outcomes and costs as well as cost-effectiveness of the 5-grass tablet vs. Grazax® and Alk Depot SQ (ALK-Abello, Hørsholm, Denmark) alongside symptomatic medication and symptomatic treatment alone for grass pollen allergic rhinoconjunctivitis [15]. In this analysis the 5-grass tablet proved to be cost-effective compared to Grazax® and Alk Depot SQ, and a symptomatic treatment. The cost-utility ratio of the 5-grass tablet vs symptomatic treatment was €14,728 per QALY; incremental costs were €1,356 and incremental QALYs 0.092. The 5 grass-tablet was the dominant strategy compared to Grazax® and Alk Depot SQ. Four further publications of which three health economic studies assessed Grazax and one Alk Depot SQ vs placebo, and concluded that Alk Depot SQ was dominant versus placebo, and Grazax® had reasonable cost-utility ratios (e.g., € 18,263 in Germany for an annual tablet price of € 1,500) [39,53,56,57].

## Conclusions

The present analysis suggests the 5-grass tablet to be cost-effective in comparison to a selected allergoid mix, mirroring a representative average of a therapeutic class in AIT. The robustness of this statement has been addressed and confirmed in extensive deterministic and probabilistic sensitivity and scenario analyses.

## Additional file

Additional file 1:
**Meta-analysis.**
