# Supplementary material for: Health economic comparison of SLIT allergen and SCIT allergoid immunotherapy in patients with seasonal grass-allergic rhinoconjunctivitis in Germany
Source: Clin Transl Allergy. 2015 Jan 21;5:1. doi: 10.1186/s13601-015-0045-z (PMC4330981; doi:10.1186/s13601-015-0045-z)
Supplement: Additional file 1: — Meta-analysis. [file 13601_2015_45_MOESM1_ESM.docx]

# Additional file 1 – Meta-analysis

For the first step of the meta-analysis, a literature review was conducted in the PubMed database to identify randomized controlled trials (RCTs) for Oralair and Allergoid Mix from March until present time (Table A1). Studies on allergoid compounds before March 2012 were included in a meta-analysis by DiBona et al. [1] and also used in the current analysis. The literature review yielded one more study for Oralair [2].

As a next step, efficacy data reported as symptom scores were extracted for the treatment arms of the included studies, along with the corresponding standard deviations and number of patients treated. If data could not be extracted from the original publication, DiBona was referenced.

Table A 1 Extracted numbers of patients, symptom scores and standard deviations of the active and placebo arm.

|  | **Population** | **Active** | | | **Placebo** | | |
| --- | --- | --- | --- | --- | --- | --- | --- |
|  |  | N | **Mean** | SD | N | **Mean** | SD |
| **Oralair** |  |  |  |  |  |  |  |
| Didier 2007 [3] | Adults | 136 | **3.58** | 2.976 | 148 | **4.93** | 3.229 |
| Wahn 2009 [4] | Children | 131 | **3.25** | 2.86 | 135 | **4.51** | 2.93 |
| Didier 2011 [5] | Adults | 149 | **2.67** | 3.63 | 165 | **4.03** | 3.71 |
| Cox 2012 [2] | Adults | 208 | **3.21** | 4.54 | 228 | **4.16** | 4.51 |
| **Allergoid Mix** |  |  |  |  |  |  |  |
| Pfaar 2012 [6] | Adults | 126 | **4.03** | 2.3 | 53 | **4.9** | 2.37 |
| Corrigan 2009 [7] | Adults | 71 | **166.5** | 114.93 | 71 | **218** | 135.39 |
| Drachenberg 2001 [8] | Adults | 74 | **0.75** | 0.44 | 50 | **0.95** | 0.41 |
| Du Buske 2011 [9] | Adults | 485 | **5.89** | 4.18 | 488 | **6.52** | 4.22 |
| Brewczynski 1999 [10] | Adults | 10 | **59.5** | 32.6 | 8 | **122.4** | 85.13 |

Symptom Scores were measured with the Rhinoconjunctivitis Total Symptom Score (RTSS) in the Oralair studies. Six different symptoms including stuffy nose, runny nose, itchy nose, sneezing, watery eyes, and itchy eyes were rated by the scoring system on a daily base within a four-point scale (0=none, 1=mild, 2=moderate, 3=severe). Thus, symptom scores vary between 0 and 18. Standardized mean difference (SMD) was used to compare data measured with different instruments.

$$SMD=\frac{M_{2}-M_{1}}{{SD}_{pooled}}$$

M1 = Mean SS Placebo, M2 = Mean SS Active, SD_pooled_ = Pooled Standard Deviation

This method is recommended by published guidelines for conducting meta-analysis [1, 11] and was applied to all individual symptom scores of the included RCTs (Table A2). The results were pooled using a fixed effect model [12, 13] to obtain a single SMD per treatment.

Table A 2 Standard mean differences (SMDs) with intervals for every study seperately and per treatment combined.

|  | **I^2** | **SMD** | **Variance** | **95 % CI Interval** | |
| --- | --- | --- | --- | --- | --- |
| **Oralair** |  |  |  |  |  |
| Didier 2007 [3] |  | -0.434 | 0.014 | -0.670 | -0.198 |
| Wahn 2009 [4] |  | -0.435 | 0.015 | -0.678 | -0.192 |
| Didier 2011 [5] |  | -0.370 | 0.013 | -0.594 | -0.147 |
| Cox 2012 [2] |  | -0.210 | 0.009 | -0.398 | -0.021 |
| ***Pooled Oralair*** | ***3%*** | ***-0.343*** | ***0.056*** | ***-0.453*** | ***-0.233*** |
| **Allergoid Mix** |  |  |  |  |  |
| Pfaar 2012 [6] |  | -0.375 | 0.027 | -0.698 | -0.052 |
| Corrigan 2009 [7] |  | -0.410 | 0.029 | -0.743 | -0.078 |
| Drachenberg 2001 [8] |  | -0.467 | 0.034 | -0.831 | -0.104 |
| Du Buske 2011 [9] |  | -0.150 | 0.004 | -0.276 | -0.024 |
| Brewczynski 1999 [10] |  | -1.025 | 0.254 | -2.013 | -0.037 |
| ***Pooled Allergoid Mix*** | ***47%*** | ***-0.236*** | ***0.054*** | ***-0.342*** | ***-0.131*** |

The use of SMD parameter is still limited in a health economic model. Thus, quality of life data and costs for transition probabilities can not be attached to the SMD. However, if SMD value is expressed as RTSS, interpretation becomes possible. This procedure was described in detail in the appendix of Westerhout et al. [14]. In order to estimate the symptom score for placebo, all placebo means (see Table 1) that were measured with the RTSS were averaged to 4.48 (Oralair studies). In conclusion, all pooled SMDs are multiplied with the typical standard deviation of 3.55 and added to the placebo symptom score. The resulting symptom scores are displayed in Table A3, final scores can be found in the method section.

Table A 3 Back-transformation of pooled SMDs to mean difference and symptom scores.

|  | **SMD** | **Mean difference** | **Symptom Score (range 0-18)** |
| --- | --- | --- | --- |
| **Oralair** | -0.343 | -1.218 | **3.26** |
| **Allergoid Mix** | -0.236 | -0.839 | **3.64** |
| **Placebo** | - | - | **4.48** |

# References

1. Di Bona D, Plaia A, Leto-Barone MS, La Piana S, Di Lorenzo G: **Efficacy of subcutaneous and sublingual immunotherapy with grass allergens for seasonal allergic rhinitis: a meta-analysis-based comparison**. *The Journal of allergy and clinical immunology* 2012, **130**(5):1097-1107 e1092.

2. Cox LS, Casale TB, Nayak AS, Bernstein DI, Creticos PS, Ambroisine L, Melac M, Zeldin RK: **Clinical efficacy of 300IR 5-grass pollen sublingual tablet in a US study: the importance of allergen-specific serum IgE**. *The Journal of allergy and clinical immunology* 2012, **130**(6):1327-1334 e1321.

3. Didier A, Malling HJ, Worm M, Horak F, Jager S, Montagut A, Andre C, de Beaumont O, Melac M: **Optimal dose, efficacy, and safety of once-daily sublingual immunotherapy with a 5-grass pollen tablet for seasonal allergic rhinitis**. *The Journal of allergy and clinical immunology* 2007, **120**(6):1338-1345.

4. Wahn U, Tabar A, Kuna P, Halken S, Montagut A, de Beaumont O, Le Gall M: **Efficacy and safety of 5-grass-pollen sublingual immunotherapy tablets in pediatric allergic rhinoconjunctivitis**. *The Journal of allergy and clinical immunology* 2009, **123**(1):160-166 e163.

5. Didier A, Worm M, Horak F, Sussman G, de Beaumont O, Le Gall M, Melac M, Malling HJ: **Sustained 3-year efficacy of pre- and coseasonal 5-grass-pollen sublingual immunotherapy tablets in patients with grass pollen-induced rhinoconjunctivitis**. *The Journal of allergy and clinical immunology* 2011, **128**(3):559-566.

6. Pfaar O, Urry Z, Robinson DS, Sager A, Richards D, Hawrylowicz CM, Brautigam M, Klimek L: **A randomized placebo-controlled trial of rush preseasonal depigmented polymerized grass pollen immunotherapy**. *Allergy* 2012, **67**(2):272-279.

7. Corrigan CJ, Kettner J, Doemer C, Cromwell O, Narkus A: **Efficacy and safety of preseasonal-specific immunotherapy with an aluminium-adsorbed six-grass pollen allergoid**. *Allergy* 2005, **60**(6):801-807.

8. Drachenberg KJ, Wheeler AW, Stuebner P, Horak F: **A well-tolerated grass pollen-specific allergy vaccine containing a novel adjuvant, monophosphoryl lipid A, reduces allergic symptoms after only four preseasonal injections**. *Allergy* 2001, **56**(6):498-505.

9. DuBuske LM, Frew AJ, Horak F, Keith PK, Corrigan CJ, Aberer W, Holdich T, von Weikersthal-Drachenberg KJ: **Ultrashort-specific immunotherapy successfully treats seasonal allergic rhinoconjunctivitis to grass pollen**. *Allergy and asthma proceedings : the official journal of regional and state allergy societies* 2011, **32**(3):239-247.

10. Brewczynski P, Kroon A: **Wirksamkeit und Verträglichkeit einer Immuntherapie mit modifizierten Gräserpollenallergenen**. *Allergologie* 1999, **22**:411-420.

11. Cochrane Collaboration: **Cochrane Handbook for Systematic Reviews of Interventions**. 2011.

12. Sutton A, Abrams K, Jones D, Sheldon T, Song F: **Methods for Meta-analysis in Medical Research**. Chichester: John Wiley & Sons; 2000.

13. Higgins JP, Thompson SG: **Quantifying heterogeneity in a meta-analysis**. *Statistics in medicine* 2002, **21**(11):1539-1558.

14. Westerhout KY, Verheggen BG, Schreder CH, Augustin M: **Cost effectiveness analysis of immunotherapy in patients with grass pollen allergic rhinoconjunctivitis in Germany**. *Journal of medical economics* 2012, **15**(5):906-917.
